# Identification of Cancer Hub Gene Signatures Associated with Immune-Suppressive Tumor Microenvironment and Ovatodiolide as a Potential Cancer Immunotherapeutic Agent

**DOI:** 10.3390/cancers13153847

**Published:** 2021-07-30

**Authors:** Jia-Hong Chen, Alexander T. H. Wu, Bashir Lawal, David T. W. Tzeng, Jih-Chin Lee, Ching-Liang Ho, Tsu-Yi Chao

**Affiliations:** 1 Graduate Institute of Clinical Medicine, College of Medicine, Taipei Medical University, Taipei 11031, Taiwan; wen17140@mail.ndmctsgh.edu.tw; 2 Division of Hematology/Oncology, Department of Medicine, Tri-Service General Hospital, National Defence Medical Center, Taipei City 114, Taiwan; Charileho22623@gmail.com; 3 The PhD Program of Translational Medicine, College of Science and Technology, Taipei Medical University, Taipei 11031, Taiwan; chaw1211@tmu.edu.tw; 4 Clinical Research Center, Taipei Medical University Hospital, Taipei Medical University, Taipei 11031, Taiwan; 5 Graduate Institute of Medical Sciences, National Defense Medical Center, Taipei 11490, Taiwan; 6 Taipei Heart Institute (THI), Taipei Medical University, Taipei 11031, Taiwan; 7 PhD Program for Cancer Molecular Biology and Drug Discovery, College of Medical Science and Technology, Taipei Medical University and Academia Sinica, Taipei 11031, Taiwan; d621108004@tmu.edu.tw; 8 Graduate Institute for Cancer Biology & Drug Discovery, College of Medical Science and Technology, Taipei, Medical University, Taipei 11031, Taiwan; 9 School of Life Sciences, The Chinese University of Hong Kong, Hong Kong; allqwdd@gmail.com; 10 Department of Otolaryngology-Head and Neck Surgery, Tri-Service General Hospital, National Defense Medical Center, 325 Cheng-Kung Road Section 2, Taipei City 114, Taiwan; doc30450@gmail.com; 11 Division of Hematology and Oncology, Department of Internal Medicine, Taipei Medical University-Shuang Ho Hospital, New Taipei City 235, Taiwan; 12 Taipei Cancer Center, Taipei Medical University, Taipei City 11031, Taiwan

**Keywords:** cancer hub, tumor microenvironments, onco-immune profiling, differentially expressed genes, ovatodiolide, cancer immunotherapy

## Abstract

**Simple Summary:**

In order to identify common genes associated with the pathology of multiple cancers, we integrated differential expressed gene (DEGs) from datasets of six cancers (liver, lung colorectal, gastric, prostate, and breast cancers) and identified six DEGs common to the six cancers. We conducted enrichment analysis and our results suggested that the DEGs are involved in the tumorigenic properties, including distant metastases, treatment failure, and survival prognosis. Notably, our results suggested high frequencies of genetic and epigenetic alterations of the DEGs in association with tumor staging, immune evasion, poor prognosis, and therapy resistance. Translationally, we intended to identify a drug candidate with the potential for targeting the DEGs. Using a molecular docking platform, we estimated that ovatodiolide, a bioactive anti-cancer phytochemical, has high binding affinities to the binding pockets of the hub genes and thus could serve as a potential drug candidate for targeting the DEGs.

**Abstract:**

Despite the significant advancement in therapeutic strategies, breast, colorectal, gastric, lung, liver, and prostate cancers remain the most prevalent cancers in terms of incidence and mortality worldwide. The major causes ascribed to these burdens are lack of early diagnosis, high metastatic tendency, and drug resistance. Therefore, exploring reliable early diagnostic and prognostic biomarkers universal to most cancer types is a clinical emergency. Consequently, in the present study, the differentially expressed genes (DEGs) from the publicly available microarray datasets of six cancer types (liver, lung colorectal, gastric, prostate, and breast cancers), termed hub cancers, were analyzed to identify the universal DEGs, termed hub genes. Gene set enrichment analysis (GSEA) and KEGG mapping of the hub genes suggested their crucial involvement in the tumorigenic properties, including distant metastases, treatment failure, and survival prognosis. Notably, our results suggested high frequencies of genetic and epigenetic alterations of the DEGs in association with tumor staging, immune evasion, poor prognosis, and therapy resistance. Translationally, we intended to identify a drug candidate with the potential for targeting the hub genes. Using a molecular docking platform, we estimated that ovatodiolide, a bioactive anti-cancer phytochemical, has high binding affinities to the binding pockets of the hub genes. Collectively, our results suggested that the hub genes were associated with establishing an immune-suppressive tumor microenvironment favorable for disease progression and promising biomarkers for the early diagnosis and prognosis in multiple cancer types and could serve as potential druggable targets for ovatodiolide.

## 1. Introduction

The current global burden of cancer was estimated to be 19.3 million cases in 2020 and is expected to be 28.4 million cases in 2040 [1], a 47% rise from 2020. In line with the previous trend [2,3], the 2020 global cancer statistic report of cancer incidences and mortality indicate that breast (11.7% and 6.9%), lung (11.4% and 18%), colorectal (10.0% and 9.4%), prostate (7.3% and 3.8%), stomach (5.6% and 7.7%), and liver (4.7% and 8.3%) cancers are the most commonly diagnosed and the leading cause of cancer mortality globally [1]. With the exception of prostate and breast cancers, which are sex-specific, the prevalence and mortality rates of these cancers combined were higher in men than in women, at 45.5% and 42.1%, respectively. These six cancers, which constituted 50.7% and 54.1% of the total global cases and cancer deaths, respectively, are the primary focus of the current study and are collectively referred to as hub cancers hereafter. Therefore, it is crucial to identify the promising early diagnostic and prognostic biomarkers that may assist in elucidating the underlying molecular mechanisms of these cancers and simultaneously improve the clinical therapeutics [4].

Microarray technology and bioinformatics analysis have become a promising and valuable tool for screening significant genetic or epigenetic variations that occur during carcinogenesis and understanding the pathogenesis of the diseases for effective diagnosis, prognosis and planning adequate therapeutic strategies [5,6,7,8]. However, the outcome from most of these findings has not yielded significant translational success in predicting reliable universal biomarkers for the diagnosis and prognosis of the hub cancers. Thus, independent analysis of microarray and RNAseq data from different cancer datasets with subsequent integration of differentially expressed genes (DEGs) across the cancer types may help identify the universal markers concordant across multiple cancer studies with increased confidence and translational relevance to the clinics. In addition to the lack of reliable and early diagnostic biomarkers, the resistance to chemotherapy and adverse effects associated with chemotherapeutic agents currently use in clinics are jointly responsible for the poor prognosis of the hub cancer patients [9,10]. Therefore, there is an urgent need to develop new, affordable, effective and safer anticancer drugs [11].

Ovatodiolide, is a phytochemical isolated from *Anisomeles indica* (L.) Kuntze [12]. This plant originates from Taiwan and is commonly known as “Fang Feng Cao” by the traditional Taiwanese herbalist where it is commonly used as an oral remedy for stomachache, swelling, abdominal pain, hypertension, arthritis, immunodeficiency disease, hepatic diseases, hemorrhoids, and arthritis [13]. The plant has been reported for various biological activities, including analgesic, anti-inflammatory, antimicrobial, hypotensive, hepatoprotective, and anti-proliferative activities [14]. Ovatodiolide has been identified as the major bioactive compound responsible for the biological activities of this plant and has been previously isolated and evaluated for anti-cancer activities against several human cancer cell lines [12]. In our previous studies, we found that ovatodiolide sensitizes aggressive human cancer cells to chemotherapy and ameliorates the cancer stemness phenotype and chemotherapy-associated toxicity in an in vitro or/and in vivo models of breast cancer [15], glioblastoma [16], oral squamous cell carcinoma [17,18], colon cancer [19], and nasopharyngeal carcinoma [20]. Studies elsewhere have also reported ovatodiolide for significant anti-cancer activities against hepatic cancer stem cells [21], renal [22], and oral [23] cancers.

Our earlier mechanistic study indicated that inhibition of numerous oncogenic molecules and signaling pathways such as Wnt/β-catenin, Hippo/YAP1, PI3K/mTOR, exosomal Mir-21/STAT3/β-Catenin, JAK2/STAT3/JARID1B, tumor necrosis factor (TNF)-α, nuclear factor (NF)-κB, matrix metalloproteinases (MMPs), and FLICE inhibitory protein (FLIP) were associated with the anti-cancer activity of this bioactive phytochemical [15,17,18,20,22]. However, the therapeutic potential of ovatodiolide against the hub genes identified in the current study via bioinformatics integrations of DEGs from the hub cancers has never been explored. Herein, our molecular docking analysis suggested that ovatodiolide docked well into the binding sites of these hub genes with estimated higher binding preferences for *IRAK3*, SEC168, and *TNPO2*; this suggested that ovatodiolide could be a drug candidate for targeting these oncogenic hub genes. In summary, the identified hub genes may provide novel insights on the early diagnosis and prognosis of hub cancers by serving as promising biomarkers/druggable targets for ovatodiolide.

## 2. Methods

### 2.1. Collection of Microarray of Cancer and Normal Samples

The microarray datasets of six cancer types (lung, liver, colorectal, prostate, gastric, and breast cancers) were collected from the NCBI Gene Expression Omnibus (GEO), a public functional genomics data repository of high throughput gene expression (http://www.ncbi.nlm.nih.gov/geo/, accessed on 11 May 2021, Figure 1). The dataset containing primary or metastatic cancer tissues (tumor samples) and normal human samples (normal counterparts) were included (Table 1). Identification of DEGs was performed using the LIMMA R package. The Benjamini–Hochberg correction method was used for *p*-value adjustment to false discovery rate (FDR). FDR < 0.05 and |logFC| > 1.5 was set as a cutoff point for DEGs selection. Online tool Multiple List Comparator (https://www.molbiotools.com/listcompare.html, accessed on 19 May 2021) was used to visualize the intersected DEGs and generate a Venn diagram for the visualization of the overlapping DEGs. In addition, we collected TCGA data of mRNA expression levels, copy number variation (CNV), single nucleotide variation (SNV), and methylation levels of the six hub genes in LUAD, BRCA, STAD, COAD, and LICH patients as well as the survival duration of these patients from the National Cancer Institute (NCI) Genomic Data Commons (GDC) (https://gdc.cancer.gov/, accessed on 27 May 2021). The distribution of the sample collection is presented in Table 2. All analyses of GDC data were conducted using the GSCLite web package.

### 2.2. Differential Expression Analysis of the Hub Genes between Tumor Stage and Molecular Subtypes

In order to predict the clinical relevance of the hub genes, we analyzed the differential expression levels of the hub genes between tumor stage and molecular subtypes. To make the subtypes analysis feasible, the number of subgroups of subtypes must have at least 10 samples. Only lung, stomach, and breast cancer met this criterion and hence were included for the subtype analysis. The RNA-Seq by Expectation-Maximization (RSEM) normalized expression values were used for the differential expression levels of the hub genes between the molecular subtypes of gastric cancer (Epstein–Barr virus (EBV), microsatellite instability (MSI), genomically stable (GS) and chromosomally unstable (CIN)), breast cancer (basal, Her2, LumA, and LumB) and lung cancer (subtypes 1–6). Analysis of variance (ANOVA) *t*-test was used for the statistical analysis. A *p*-value of less than 0.05 was considered significant. 

### 2.3. Prognosis Analysis of the Hub Genes

We queried the associations between the gene expression profile and prognosis of the cohorts by integrating tumor gene expression levels of the hub genes and overall survival data from the patients of each of the hub cancers using the PREdiction of Clinical Outcomes from Genomic profiles (PRECOG) server (https://precog.stanford.edu/index.php, accessed on 29 May 2021). Similarly, we queried the survival differences between the mRNA expression levels of each hub gene across the combined cohorts of the hub cancers using the Gene Expression Profiling Interactive Analysis (GEPIA) webtool. Kaplan–Meir plots were used to visualized the survival differences between hub cancer cohorts with high and low gene expression levels.

### 2.4. Functional Enrichment Analysis of the Hub Genes

Online databases (https://maayanlab.cloud/Enrichr/, accessed on 29 May 2021) were used for the gene ontology function and KEGG (Kyoto Encyclopedia of Genes and Genomes) pathway enrichment analyses of the DEGs. The GO and KEGG terms with FDR < 0.05 were regarded as significant functions and pathways and were visualized using the R package cluster Profiler.

### 2.5. Gene–Gene and Protein–Protein Interaction Network Construction Analysis

Network construction for gene–-gene interaction (GGI) and protein–protein interaction (PPI) of the hub genes were performed via the GENEMANIA (https://genemania.org/, accessed on 1 June 2021) and the Search Tool for the Retrieval of Interacting Genes (STRING; http://string.embl.de/, accessed on 23 May 2021), respectively. The PPIs of the DEGs were constructed with a confidence score of 0.70.

### 2.6. Analysis of Single Nucleotide Variation (SNV) and Copy Number Variation (CNV) of the Hub Genes

We collected SNV data of seven variant types of effective mutations (Missense_Mutation, Nonsense_Mutation, Frame_Shift_Ins, Splice_Site, Frame_Shift_Del, In_Frame_Del, In_Frame_Ins) of lung, breast, colon prostate, gastric, and liver cancer from the NCI Genomic Data Commons (https://gdc.cancer.gov/, accessed on 29 May 2021), and analyzed the frequencies and occurrence of these SNV in the hub genes across the six hub cancers via the GSCALite (http://bioinfo.life.hust.edu.cn/web/GSCALite/, accessed on 29 May 2021) server [24]. SNV percentage of each gene’s coding region was calculated by: Num of Mutated Sample/Num of Cancer Sample. Mutation Annotation Format (MAF) tools were used for data visualization and to generate the waterfall plot [25]. The CNV data were processed with GISTICS2.0 [26]. The frequency of four types of CNV—Hete Amp: heterozygous amplification (CNV = 1), Hete Del: heterozygous deletion, (CNV = −1), Homo Amp: homozygous amplification (CNV = 2), and Homo Del: homozygous deletion (CNV = −2)—in the six hub genes across the six cancer types were visualized. The SNV or CNV data and clinical overall survival data were combined and analyzed for survival differences between mutated and non-mutated genes (SNV) and between CNV using R package survival. A log-rank test [27] was also performed to compare the distributions of groups while Cox regression [28] was performed to estimate the hazards. A *p*-value < 0.05 was considered as significant.

### 2.7. Methylation Analysis of the Hub Genes

We used Pearson’s product–moment correlation coefficient and followed a t-distribution to analyze the correlation between the mRNA expression and methylation levels of the hub genes across the six cancer types. The *p*-value was adjusted by FDR and genes with FDR ≤ 0.05 were considered as significant. In addition, we collected the clinical overall survival data of the cohorts and analyzed the survival differences between hyper and hypo-methylation levels of the hub genes across the six cancers. A log-rank test was also performed to compare the distributions of two groups while Cox regression analysis was conducted to estimate the hazards (risk of death). A *p*-value < 0.05 was considered as significant.

### 2.8. Analysis of the Hub Genes Expressions Correlation with Gene Expression Profiles Suggestive of Immune and Immuno-Suppressive Cell Infiltrations

We used the Tumor Immune Estimation Resource (TIMER2.0) (http://timer.cistrome.org/, accessed on 21 May 2021) [29] to analyze the hub gene expression correlations with the levels of gene expression suggestive of immune infiltrations (B Cell, CD8+ T Cell, CD4+ T Cell, macrophage, neutrophil, and dendritic cells) in the six cancer types. We also analyzed the hub gene expression correlations with the gene expression profiles suggestive of immunosuppressive cells infiltration. Four immunosuppressive cells that are known to promote T cell exclusion—vis myeloid-derived suppressor cell (MDSCs), cancer-associated fibroblast (CAF), tumor-associated macrophages (M2-TAM), and regulatory T cell (Treg)—were included. The correlation analysis was conducted using the purity-corrected partial Spearman’s rho value and statistical significance (*p* < 0.05). We used GraphPad Prism Software (version 8.0.0 for Windows) for data visualization. Heat maps were used to visualize the correlations between the hub genes expression levels and the gene expression profiles suggestive of immune/immunosuppressive cell infiltrations across the six cancers. In addition, we used the QUERY module of the TIDE algorithm to evaluate the correlations between the hub gene expression and levels of gene expression suggestive of T cell exclusion and dysfunctional T cell phenotypes of the six hub cancers [30]. 

### 2.9. Gene Prioritization Analysis of The Hub Genes

We access the gene prioritization of the hub genes across two parameters, namely the response to ICB therapy and gene knockout phenotype in CRISPR screens. The z-score in the Cox-PH regression was used to evaluate the effect of the gene on the expression on patient survival in ICB treatment cohorts. The normalized logFC in CRISPR screens was employed to evaluate the effect of gene knockout on lymphocyte-induced tumor death in cancer models [30].

### 2.10. Drug Response and Sensitivity Analysis of the Hub Genes

We used the Spearman correlation analysis to explore the correlation between mRNA expression levels of the hub genes and IC_50_ concentrations of small molecules against cancer cell lines in the Therapeutics Response Portal (CTRP) database. In addition, we also analyzed the correlation of the expression levels of the hub genes with sensitivity to chemotherapy of the cancer patient using the ROC Plotter (http://www.rocplot.org/, accessed on 28 May 2021) algorithm, a transcriptome-based tool for predictive biomarkers by linking gene expression and response to therapy in cancer patients [31].

### 2.11. Molecular Docking Studies

The molecular docking study of the hub genes with ovatodiolide was conducted using AutoDock VINA (version 0.8) [32] software according to the protocols described in previous studies [33,34]. The three-dimensional (3D) structure of the hub genes *RAB31* (PDB:2fg5), *IRAK3* (PDB:6ruu), *OBSCN* (PDB:2e01), *LIN9* (PDB:6c48), *TNPO2* (PDB:2z5j), and *SEC16B* (PDB:3mzk) were downloaded from the protein data bank (PDB). The 3D structure of the ovatodiolide was obtained in the Sybyl mol2 format using the Avogadro molecular builder and visualization tool version 1.XX [35] before subsequently being transformed into the protein data bank (PDB) format using the PyMOL Molecular Graphics System, version 1.2r3pre. The PDB files of the crystal structures of the receptors (hub genes) were transformed to pdbqt format by using AutoDock VINA (version 0.8) [32]. The removal of water molecules and the addition of Kolman charges and hydrogen atoms were carried out before docking simulation [36]. Visualization and analysis of the docked complex were performed using the PyMOL software and Discovery studio visualizer (version 19.1.0.18287, BIOVIA, San Diego, CA, USA) [37].

## 3. Results

### 3.1. RAB31/IRAK3/OBSCN/LIN9/TNPO2/SEC16B Are Hub Genes Associated with the Development of Breast, Lung, Colorectal, Liver, Prostate, and Stomach Cancers (Hub Cancers)

**Figure 2:**
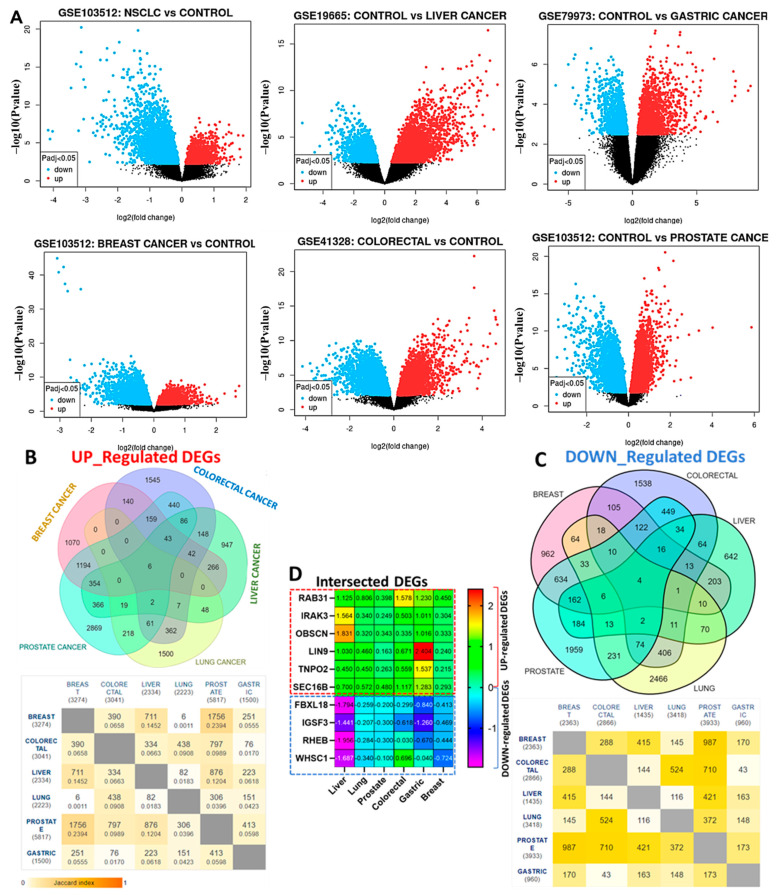
Identification of differentially expressed genes (DEG) between the cancer patients and healthy controls. (**A**) Volcano plot showing the distribution of DEGs between the cancer patients and healthy controls. The top DEGs are represented satisfying the criteria of logFC value and *p* < 0.05. The color indicates high expression (red) and low expression (blue). (**B**) The Venn diagrams of the overlapping up-regulated and (**C**) down-regulated DEGs in the six cancers. (**D**) Heat showing the logFC distribution of the 10 overlapping DEGs (6 up-regulated and 4 down-regulated genes) in all six GEO datasets.

The detailed information for the GEO datasets for each of the six cancer types is shown in Table 1 while the distribution of DEGs in the six cancer types analyzed are represented by the volcano plots (Figure 2A). Overall, we identified 3083 DEGs in colorectal (1545 up-regulated and 1538 downregulated), 2032 DEGs in breast (1070 up-regulated and 962 down-regulated), 4825 DEGs in prostate (2869 up-regulated and 1959 down-regulated), 3966 DEGs in lung (1500 up-regulated and 2466 down-regulated), 1589 DEGs in liver (947 up-regulated and 642 downregulated), and 2460 DEGs in gastric cancer (1499 up-regulated and 961 downregulated) (Figure 2B,C). We further identified six overlapping up-regulated DEGs, *RAB31, IRAK3, OBSCN, LIN9, TNPO2,* and *SEC16B*, while four down-regulated genes were obtained after the integrated analysis of six GEO datasets (Figure 2D).

### 3.2. The Hub Genes Expressions Are Associated with Clinical Prognosis of the Hub Cancer Patients

**Figure 3:**
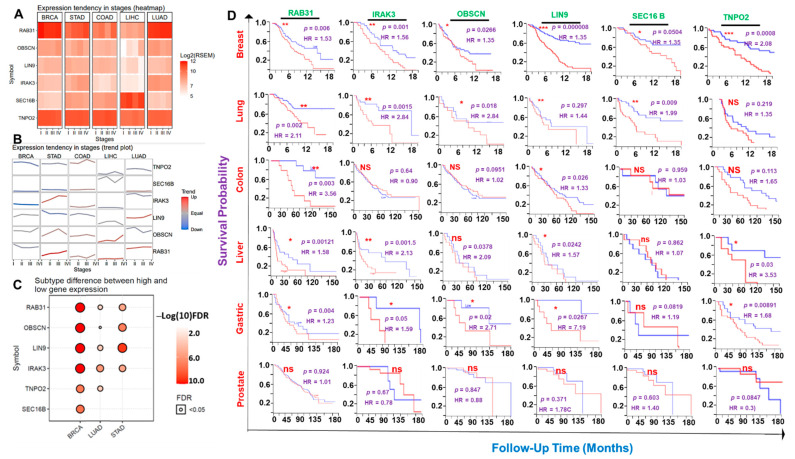
The cancer hub gene signatures are of clinical prognostic relevance. (**A**) Heatmap and (**B**) trend plot showing the differential expression levels of the hub genes between tumor stages across the six cancers. (**C**) Bubble plot showing significant differences in the expression levels of the hub genes in the molecular subtypes of the breast, lung, and stomach cancers. (**D**) Kaplan–Meir plot showing survival differences between hub cancers cohorts with high and low gene expression levels. * *p* < 0.05; ** *p* < 0.01; *** *p* < 0.001. HR: Hazard ratio, ns: non-significant.

Our differential expression analysis suggested that the expression levels of the hub genes vary with the molecular subtypes of the breast, lung, and stomach cancers and, in most cases, elevates with increased tumor stages of the six cancer hub (Figure 3A–C, Figure S1). Notably, we found that the six hub genes were significantly (all *p*-value < 0.05) associated with shorter survival duration of the breast cancer cohorts while none of the genes was significantly associated (all *p* > 0.05) with a prognosis of prostate cancer cohorts (Figure 3D). Furthermore, with the exception of *TNPO2* in lung cancer, *OBSCN* in liver cancer, and SEC16 in gastric and colon cancers, the high expression levels of the hub genes predicted a worse prognosis of the patients. Similarly, we queried the survival differences between the mRNA expression levels of each hub gene across the combined cohorts of the hub cancers and found that higher mRNA expression levels of *RAB31*, *IRAK3*, *SEC16B*, and *LIN9* predicted shorter survival durations of the hub cancer cohorts. However, our results predicted that the mRNA expression levels of *SEC16B* and *TNPO2* had no survival significance to the cohorts (Figure S2).

### 3.3. Gene–Gene and Protein–Proteins Interactions, and Functional Enrichment of the Hub Genes

**Figure 4:**
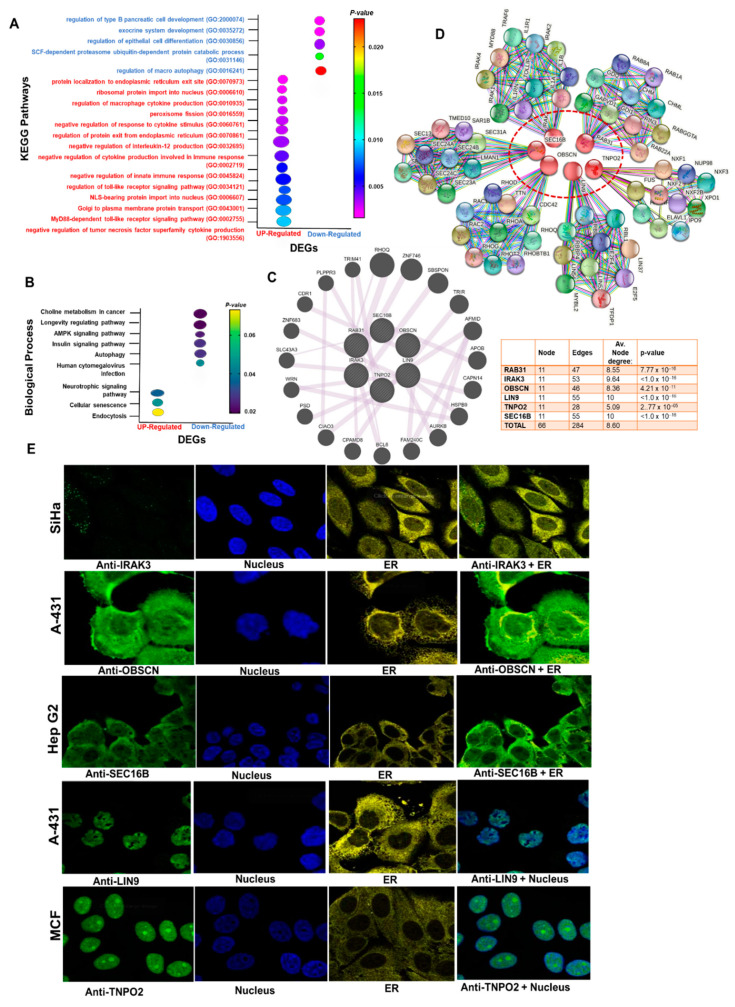
Enrichment and interaction analysis of the DEGs. (**A**) Bubble plot of KEGG pathway enrichment analysis of DEGs. (**B**) Bubble plot of GO biological enrichment analysis of DEGs. GO and pathways enrichment is ranked by their *p*-value. KEGG, Kyoto Encyclopedia of Genes and Genomes. (**C**) Cycle plot of the DEGs’ (**C**) gene–gene and (**D**) protein–protein interaction network (PPI). Node sizes are based on the degree of connectivity of the nodes. (**E**) Immunofluorescence stain of the subcellular distribution of the hub genes within the nucleus and ER of cancer cells. Gene localizations were detected based on immunofluorescence microscopy of HPA database using the following antibodies: *IRAK3* (#HPA043097), *OBSCN* (#HPA021186), *LIN9* (#HPA030241), *TNPO2* (#HPA071498), *SEC16B* (#HPA054292).

To predict the biological roles of the identified DEGs, we conducted GO and KEGG pathway enrichment analyses. In terms of the KEGG pathways, the up-regulated genes were enriched in cellular senescence and neurotrophin signaling pathways (Figure 4A). These DEGs were, however, significantly enriched in multiple biological processes related to cellular protein trafficking, regulation of cellular immune responses, and immune system evasion (Figure 4B and Table S1). Our KEGG pathway enrichment analysis predicted that the down-regulated genes mainly participated in longevity-associated signaling pathways, including autophagy, AMPK, and insulin signaling pathways, while the enriched biological functions of the downregulated DEGs were autophagy, exocrine system development, and protein ubiquitination. Analysis of gene–gene interaction suggested that these six gene sets form a gene co-expression network interaction with known oncogenes including *TRIM41, RHOQ, ZNF746, SBSPON, TRIR, AFMID, APOB, CAPN14, HSPB9, AURKB, PLPPR3, CDR1, ZNF683, SLC43A3, WRN, PSD, CPAMD8, BCL6*, and *FAM24OC* (Figure 4C), while the PPI network of up-regulated DEGs consists of 66 nodes and 284 edges with a mean node degree of 8.60 (Figure 4D). Our further analysis of gene and pathway interaction suggested a high probability of inhibition of DNA damage response and apoptosis while activating *RAS/MAPK*, RTK, EMT, and *PI3K/AKT* signaling by the hub genes (Figure S3).

To further characterize the hub genes, we acquired the experimental evidence about the sub-localization of *RAB31, IRAK3, OBSCN, LIN9, TNPO2,* and *SEC16B* in human cell lines via the Human Protein Atlas database. Our results suggested that *IRAK3* and *OBSCN* are localized to the vesicle and plasma membrane, respectively, *SEC16B* is localized to ER and plasma membrane, and *LIN9* and *TNPO2* are localized to the nucleoplasm (Figure 4E).

### 3.4. Distribution and Effect of Genetic and Epigenetic Alteration of the Hub Genes in the Hub Cancers

**Figure 5:**
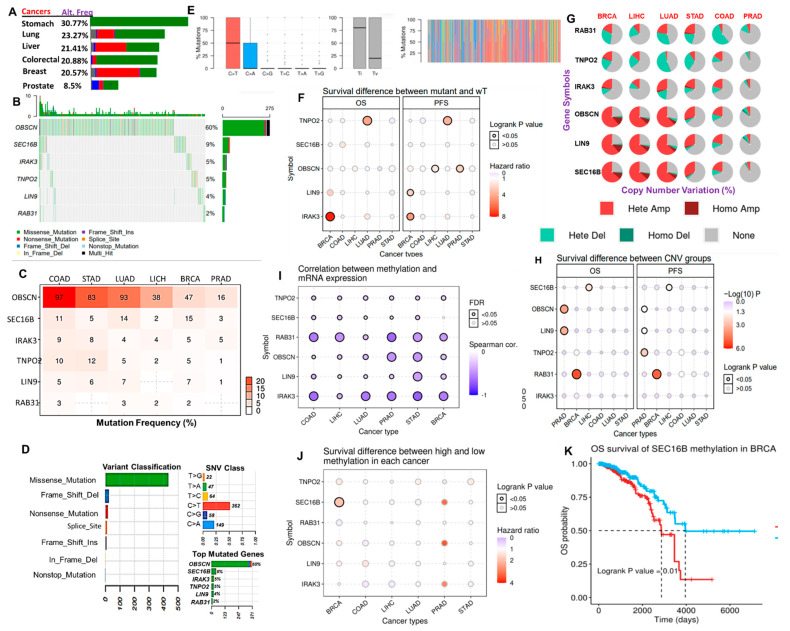
Genetic and epigenetic alterations of the cancer hub genes across the cancer types. (**A**) The bar plot showing the distribution of genetic alteration frequency of the hub gene set across the six cancer types. (**B**) The waterfall plot showing the mutation distribution of the hub genes in the hub cancers. (**C**) Bar plot showing the percentage mutation frequency of each of the genes across the six cancer types. (**D**) The summary plot of the count and type of variants in the sample and gene levels. Plot 1 (Variant Classification) shows the count of each type of effective mutation. Plot 2 (SNV class) shows the count of each SNV class of the hub gene set in the six cancer types. Plot 3 (top mutated genes per sample) shows the top mutated genes. (**E**) The Titv plot showing the distribution of transitions (Ti; A <-> G and C <-> T) and transversions (Tv) mutations of the hub gene set in the hub cancers. (**F**) The bubble plot showing the patient survival differences between the mutant and wild type of the six gene hub in the six cancer types. (**G**) A pie plot showing the proportion of different types of CNV of each of the genes in each cancer. (**H**) Bubble plot of the survival difference between each gene CNV (amplification, deletion, and wide type). The color from blue to red and the bubble size represent the statistical significance. The black outline border indicates Log-rank *p* < 0.05. (**I**) Bubble plot showing the correlation between methylation and mRNA gene expression. The blue bubbles represent negative correlations, while the bubble size represents statistical significance. (**J**) The bubble plot and (**K**) Kaplan–Meir curve showing the single gene survival result between cohorts with high and low methylation levels of the hub genes; the color from blue to red correlates with the hazard ratio and the bubble size represents statistical significance. The black outline border indicates Log-rank *p* < 0.05.

Our analysis of the frequencies of genetic alterations of the hub genes from the TCGA cancer cohorts suggested that the genetic alterations of the hub genes occur at frequencies of 30.77%, 23.27%, 21.41%, 20.88%, 20.57%, and 8.5% in stomach, lung, liver, colorectal, breast, and prostate cancer cohorts, respectively (Figure 5A). Specifically, single nucleotide variation (SNV) of *OBSCN* is the most frequently predicted gene alteration, while SNV of *SEC16B*, *IRAK3*, *TNPO2*, *LIN9*, and *RAB31* constituted 9%, 5%, 5%, 4%, and 2% of genetic alterations in the TCGA cohort of the hub cancers (Figure 5B–D). In addition, our results also suggested that the missense mutation is the most frequent effective mutation of the hub genes, having the occurrence counts of >70%, while other effective mutations including the nonsense, frame_shift_ins, splice_site, frame_shift_del, in_frame_del, and in_frame_ins constituted a smaller proportion of the SNV of the gene hub in the TCGA samples of the hub cancers (Figure 4D, Table S1). The majority of the mutations were predicted to be C > T and T > C transitioned, and C > G and C > A transversion (Figure 5E). However, our survival analysis indicated that SNV of *TNPO2* and *IRAK3* are associated with a worse prognosis of LUAD and BRCA cohorts. *OBSCN* predicted a poor prognosis of LIHC and PRAD while LIN is associated with poor survival of BRCA cohorts (Figure 5F). Our correlation analysis also suggested that the copy number alterations of the hub genes occur most frequently in BRCA, LIHC, and LUAD, while the least CNA occurs in PRAD. These CNA were mostly heterozygous amplification and heterozygous deletion. Our results predicted distinct types of CNA of the hub genes; *OBSCN*, *LIN9*, and *SEC16B* were predicted to exhibit amplification (heterozygous and homozygous) type of CNA while *RAB31*, *TNPO2*, and *IRAK3* were predicted to exhibit heterozygous deletion and amplification types of CNA (Figure 5G). Despite the low frequencies of CNV of the hub genes in PRAD, survival analysis predicted a shorter survival duration for those cohorts having CNV of *OBSCN* and *LIN9* (Figure 5H). CNA of *RAB31* and *SEC16B* predicted poor prognosis of BRCA and LICH, respectively. With the exception of *SEC16B* in BRCA, the methylation levels of the six hub genes were in negative correlation (all cor < 0 and all *p* < 0.05) with the mRNA expression levels in the six cancer types (Figure 5I). Surprisingly, only the methylation of SEC116B in BRCA predicted a significant (*p* < 0.05) poor prognosis of the cohorts (Figure 5J,K).

### 3.5. The Hub Genes Expressions Are Associated with the Gene Expression Profiles Suggestive of Tumor Immune Evasion 

**Figure 6:**
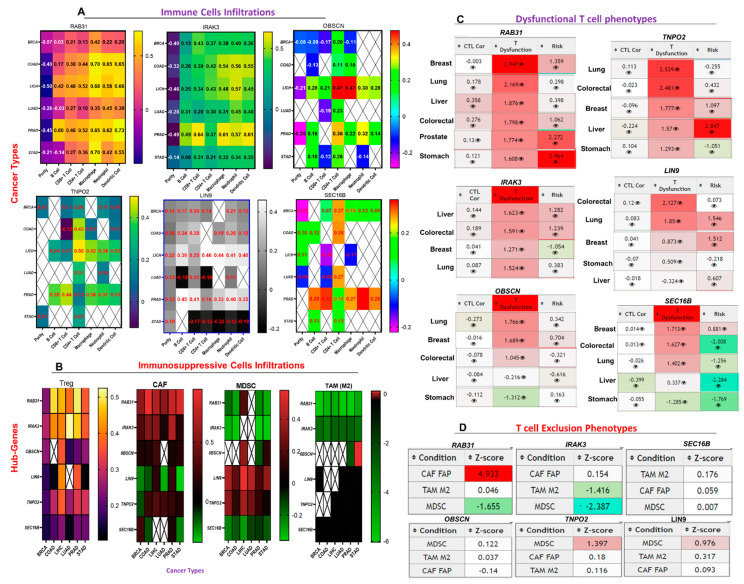
The hub genes expression correlate with the gene expression profiles suggestive of tumor immune evasion. Heat map showing the correlation between the expression levels of the six hub genes with the levels of gene expression suggestive of (**A**) six immune cells (B cell, CD8+ T Cell, CD4+ T Cell, macrophage, neutrophil and dendritic) infiltration, and (**B**) four immunosuppressive cell types, myeloid-derived suppressor cell (MDSCs), cancer-associated fibroblasts (CAF), tumor-associated macrophages (M2-TAM), and regulatory T cell (Treg) infiltration across the hub cancers. (**C**) Heat map showing the correlation between the expression levels of the six hub genes with the level of active cytotoxic lymphocyte and dysfunctional T cell phenotypes of the six hub cancer cohorts. (**D**) Summary heat map of the association between the gene expression levels and T cell exclusion phenotypes using data from 3 immunosuppressive cells. Z-score (T cell exclusion score); The interaction coefficient “(d)” of the gene divided by its standard error. Condition; T cell exclusion signature.

We assessed the correlation between the expression levels of the hub genes and the gene expression profiles suggestive of B cell, CD8+ T Cell, CD4+ T Cell, macrophage, neutrophil, and dendritic cell infiltrations across the hub cancers. We found that in all the six cancer types analyzed, the expression levels of *RAB31* and *IRAK3* were positively correlated (all *p*-value < 0.05) with the gene expression profiles suggestive of infiltrations of all immune cell types analyzed (except B cells) (Figure 6A). The degree of correction between the expression level of *RAB31* and gene expression profiles suggestive of immune infiltration were weak to strong (r = 0.128–0.452) in breast and lung cancers, and strong to very strong (r = 0.38–0.70) in colorectal, liver, stomach, and prostate cancers. *IRAK3* correlated strongly with the gene expression profile suggestive of the six immune cell infiltrations in all the cancer types, while a strong correlation between the expression of *OBSCN*, *LIN9*, *TNPO2*, and *SEC16B* and the levels of gene expression suggestive of immune infiltration was observed for prostate cancer. *LIN9* and *TNPO2* expressions correlated strongly with the gene expression profiles suggestive of immune infiltrations in LICH, while no significant association was predicted between the expressions of other genes and the levels of gene expression suggestive of immune infiltration in LICH cohorts (Figure 6A, Table S2). In all the six cancers, the expression levels of the hub genes were strongly correlated with gene expression profiles suggestive of macrophage infiltration, while a lesser or negative correlation with the levels of the gene expression profiles suggestive of B-cell infiltrations was recorded.

In addition, we assessed the correlation between the expression levels of the hub genes and the gene expression profiles suggestive of immunosuppressive cells (MDSCs, CAF, M2-TAM, and Treg) infiltrations. Surprisingly, we observed a strong to very strong correlation between the expression levels of *RAB31* and *IRAK3*, and the gene expression profiles suggestive of Treg infiltration, while a very strong to excellent correlation was observed with the levels of the gene expression profiles suggestive of CAF infiltrations in the hub cancers. The expression levels of *OBSCN*, *LIN9*, *TNPO2*, and *SEC16B* correlates positively with the gene expression profiles suggestive of Treg infiltration. TNP02 and *OBSCN* expressions were weakly correlated, while *LIN9* expression correlates negatively with the gene expression profiles suggestive of CAF infiltration in the hub cancers. *SEC16B* expression was weakly correlated with the gene expression profiles suggestive of CAF infiltration of BRCA and PRAD, and inversely correlated with the gene expression profiles suggestive of CAF infiltration in COAD and STAD. In sharp contrast with the correlation between the expression levels of the hub genes and the gene expression profiles suggestive of M1 macrophages infiltration, we observed that the expressions of the hub genes were inversely correlated with the gene expression profiles suggestive of M2-TAM infiltrations in the six cancer types. Similarly, the expression levels of *RAB31*, *IRAK3*, and *SEC16B* were inversely correlated, while *TNPO2* and *LIN9* were positively correlated with gene expression profiles suggestive of MDSCs infiltration in the six cancer types (Figure 6B, Table S3).

In order to summarize the correlation between the hub gene expression level and the gene expression profiles suggestive of the immune/immunosuppressive cell infiltrations, we queried the associations between the expression levels of these hub genes and the gene expression profile suggestive of CTL, dysfunctional T cell phenotypes, and T cell exclusion in the hub cancers. Interestingly, our results suggested that the expression levels of the hub genes were inversely correlated with the levels of gene expression suggestive of CTL infiltration in the six cancers (Figure 6C).

The high expression levels of *RAB31*, *IRAK3*, and *TNPO2* were strongly correlated with gene expression profiles suggestive of dysfunctional T cell phenotypes in the six cancers. High expression levels of *OBSCN* correlated with gene expression profiles suggestive of dysfunctional T cells only in lung and breast cancers, and *LIN9* only in colorectal and lung cancers, while the expression of *SEC16B* correlates with gene expression profiles suggestive of dysfunctional T cells in breast, lung, and colorectal cancers (Figure 6C). Furthermore, our analysis suggested that the expression of *RAB31* could be associated with the gene expression profiles suggestive of T cell exclusion phenotypes via cancer-associated fibroblast while the expression levels of *TNPO2* and *LIN9* could be associated with the gene expression profiles suggestive of T cell exclusion phenotypes via MDSC (Figure 6D).

### 3.6. The Hub Genes Expressions Predicted Chemo- and Immune Checkpoint Blockade Therapies Resistance

**Figure 7:**
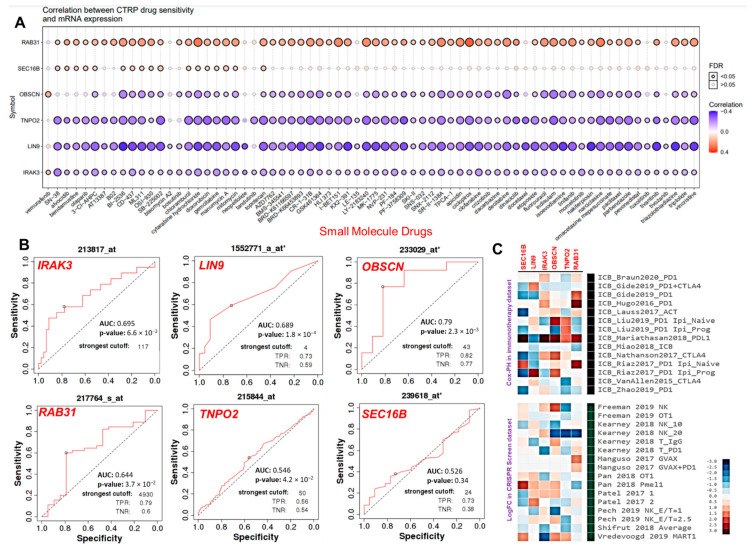
The cancer hub genes are associated with poor response to chemotherapy and immune checkpoint blockage therapy outcome. (**A**) Bubble plot of the correlation between mRNA expression levels of the hub genes and CTRP drug sensitivity. The color from blue to red represents the correlation between mRNA expression and the IC50 value of the small molecule drugs. The positive correlation means that the gene’s high expression is resistant to the drug, and vice versa. The bubble size positively correlates with the FDR significance. The black outline border indicates FDR < 0.05. (**B**) The ROC plotter of the association between the gene expression and sensitivity to chemotherapy in the hub cancer. (**C**) Heat map depicting the association between the hub genes with lymphocyte-mediated tumor killing in CRISPR screens and outcome in ICB sub cohort.

The results of the present study suggested a significant correlation between the mRNA expression levels of the hub genes and IC_50_ values of 70 small molecules. Interestingly, high mRNA expression levels of *RAB31* and *SEC16B* predicted cancer cell lines resistant to 62 and 17 small molecule anticancer drugs, respectively. The mRNA expression levels of *OBSCN* and *IRAK3* were associated with resistance to vemurafenib, while the IC_50_ concentrations of other drugs correlate negatively with the expression levels of the hub genes in the cancer cell lines (Figure 7A). In addition, we link the expression levels of the hub genes and response to anti-cancer therapy using transcriptome-level data of the cancer patients. Interestingly, our results suggested that *IRAK3*, *RAB31*, *LIN9*, and *OBSCN* has strong prognostic power with estimated AUC values of 0.695, 0.644, 0.689, and 0.79, respectively. The expression level of *TNPO2* predicted week prognostic power for clinical utility (AUC = 0.546) while the expression levels of *SEC16B* predicted no significant association with chemotherapy sensitivity of the hub cancers (Figure 7B). Finally, we access the gene prioritization of hub genes in order to predict a generalized role of each gene’s associations with ICB response outcome and phenotypes in genetic screens (CRISPR screens). Our results suggested that high gene expression levels of the hub genes are associated with resistance to worse patient outcomes to PD1, CTL4A, and PDL1 immunotherapies in the immune checkpoint blockage datasets (Figure 7C, upper panel). The prioritization of the hub genes in the gene expression profiles suggestive of tumor immune evasion and immunotherapy response occurs in the order of *RAB31* > *TNPO2* > *OBSCN* > *IRAK3* > *LIN9* > *SEC16B* (Figure 7C).

### 3.7. The Cancer Hub Genes Are Potentia Druggable Targets of Ovatodiolide

**Figure 8:**
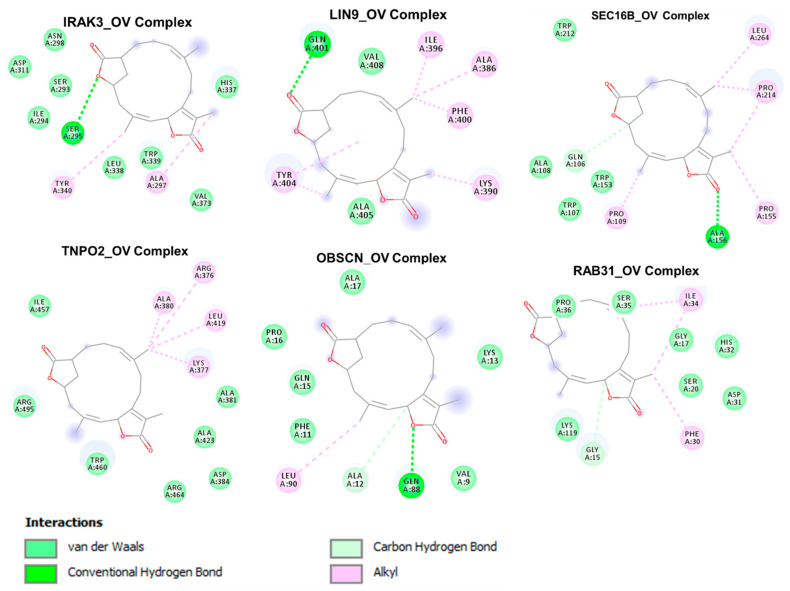
Two dimensional (2D) representation of the docking profile of ovatodiolide (OV) with the crystal structures of the cancer hub genes *RAB31* (PDB:2fg5), *IRAK3* (PDB:6ruu), *OBSCN* (PDB:2e01), *LIN9* (PDB:6c48), *TNPO2* (PDB:2z5j), and *SEC16B* (PDB:3mzk)**.** Each complex represents the interaction of the best docking pose between the receptor and the ligand.

We analyzed the potential druggability of the hub genes by ovatodiolide, an anti-cancer phytochemical identified by our group previously [17,19]. Hence, we performed a molecular docking simulation for ovatodiolide and the cancer hub genes. Our results suggested that ovatodiolide has high possibilities of interacting with the crystal structure of the hub genes *RAB31* (PDB:2fg5), *IRAK3* (PDB:6ruu), *OBSCN* (PDB:2e01), *LIN9* (PDB:6c48), *TNPO2* (PDB:2z5j), and *SEC16B* (PDB:3mzk) with estimated binding affinities of −6.5, −8.4, −6.2, −6.5, −8.1, and −8.5 Kcal/mol, respectively. Our analysis of the interactions between the hub genes (receptors) and the ligand (ovatodiolide) suggested that ovatodiolide interacts with the hub genes by several hydrogen bonding and alky interactions. The receptors and ligand complexes were predicted to be further stabilized by various Vander wall forces around the ovatodiolide backbone with the respective amino acid residue of the receptors. Several hydrophobic contacts were predictively observed between the hub genes and ovatodiolide. The predicted interactions and binding affinities suggested that *IRAK3*, SEC168, and *TNPO2* were likely the most favored receptors for ovatodiolide (Figure 8, Table 3).

## 4. Discussion

In the current study, six up-regulated DEGs were universally identified via integrated analysis of datasets of the six hub cancers. The identification of these DEGs in these hub cancers is suggestive of their concrete contribution in tumor-driving events and potential association with tumor progression and metastasis. These hub genes are likely to hold significance in the genesis of the hub cancers and might be prioritized for common biomarker detection among different cancers. The KEGG enrichment analysis suggests that the DEGs aid in cellular senescence and neurotrophin signaling pathways. The DEGs were, however, significantly enriched in multiple biological processes related to cellular protein trafficking, regulation of cellular immune responses, and immune system evasion, which are critical parameters involved in the tumor progression, metastatic progression, and treatment failure, and a major cause of patient death. Similarly, our analysis of gene and pathway interaction suggests a high probability of inhibition of DNA damage response and apoptosis while activating RAS/MAPK, RTK, EMT, and PI3K/AKT by the hub genes. RAS/MAPK, RTK, EMT, and PI3K/AKT signaling pathways are considered master regulators of normal physiological processes and their hyper-activation has been significantly correlated with growth, proliferation, metastasis, and drug resistance across various human cancers [34,38]. Furthermore, our enrichment analysis suggested that the down-regulated DEGs mainly participated in longevity-associated signaling pathways, such as autophagy, AMPK, and insulin signaling pathways. Thus, both GO and pathway enrichments suggest the involvement of the DEGs in immune invasion and migratory events of cancer development and the shorter lifespan of the cohorts. Subsequently, the six up-regulated DEGs were explored for tumor stage and subtypes expressions, and prognostic analysis, using the transcriptome data from TCGA, found that the DEGs correlated with differential expression in the molecular subtypes of the breast, lung, and stomach cancers and were significantly associated with worse overall survival of the liver, breast, lung, colorectal, and stomach cancers. Collectively, these findings suggest the significance of the hub genes as a universal biomarker signature for the six major cancer types. More importantly, our results suggest that these hub genes could serve as both early diagnostic and prognostic indicators for the six major cancer types.

The tumor microenvironment consists of tumor cells, stromal cells, and the infiltrating immune cells [39]. Immunotherapy has revolutionized the treatment of cancers, but the mechanisms that regulate immunity in the TME are complex and require more investigation. The connections among these cancer hub genes and their roles within the TME, therapeutic responses, and patient survival were predicted in this study. Our results suggest that the expression levels of *RAB31, IRAK3, OBSCN, LIN9, TNPO2,* and *SEC16B* were positively associated with the levels of the gene expression suggestive of B cell, CD8+ T Cell, CD4+ T Cell, macrophage, neutrophil, and dendritic cell infiltrations. Consistently, the gene expression profiles suggestive of immune infiltration appeared to be a predictor of dysfunctional T cell phenotypes and a worse prognosis of the cohort of those cancers. In agreement with our findings, previous studies indicated that NK (resting and activated) cell, monocyte, resting mast cell, neutrophil, M1 and M2 TAM, activated dendritic cell, CD4+ T cell, and CD8+ T cell infiltrations were significant predictors of disease relapse, even after accounting for known prognostic indicators, including adjuvant therapy [40].

The functional differences between M1- and M2-polarized tumor-associated macrophages (TAMs) play essential roles in tumorigenesis and therapeutic development [41]. Tumors are likely to contain macrophages in any of these states. The M1 and M2 TAMs are associated with distinct immunomodulatory roles; they represent extremes of a spectrum of functional states rather than truly distinct cell types [41]. The present study suggested a strong positive association between the expression levels of the hub genes and the gene expression profiles suggestive of M0 macrophages and a negative association with M2 subtypes in the six cancers. Furthermore, the hub genes expression signatures of M2 TAMs are inversely associated with gene expression profiles suggestive of T cell exclusion phenotypes (Figure 6D), indicating that the hub genes are unlikely to play a role in macrophages dependent regulation of T cell exclusion phenotypes in the hub cancers. Conversely, the gene expression profiles suggestive of dysfunctional T cell phenotypes and high survival risk (Figure 6C) predicted in cohorts with high expression levels of the hub genes suggest the oncogenic role of Mo macrophages in these cancers, a conclusion supported by previous studies [40,42].

We found that the expression levels of the hub genes positively correlated with the gene expression profiles suggestive of CAF and Treg infiltration in all the cancers. Previous experiments have shown that tumors attract Tregs to evasion of the anti-cancer immune response [43,44]. Our findings suggested that the six hub genes are likely to be associated with the regulation of Treg infiltration and supported the tumor-promoting role of Tregs, which facilitated T cell exclusion. CAFs inhibit T cell expansion by promoting the secretion and expression of immune checkpoint molecules and consequently impede anti-tumor immune response [45]. The strong correlation between the expression levels of *RAB31*, *IRAK3*, and *OBSCN* and the gene expression profile suggestive of CAF infiltrations strongly supported the tumor-promoting role of these genes in the hub cancers. However, the negative association between the levels of *LIN9* and *SEC16B* and the gene expression profile suggestive of CAFs infiltrations of the hub cancers suggested that the hub genes are likely to be involved in the regulation of tumor immune evasion via different mechanisms.

Genes highly expressed in tumor cells were expected to have positive associations with tumor purity, while those highly expressed in the tumor microenvironment are in negative associations with tumor purity [46]. Notably, *RAB31, IRAK3, OBSCN, LIN9, TNPO2,* and *SEC16B* expressions were inversely correlated with the gene expression profiles suggestive of tumor purity of the hub cancer. Based on these findings, we proposed that *RAB31, IRAK3, OBSCN, LIN9, TNPO2,* and *SEC16B* are mainly expressed in the TME rather than in the tumor cells; our results suggested that these cancer hub genes are associated with the regulation of infiltration of immune and immunosuppressive cells from the tumor microenvironment into the tumor tissues.

In addition, our results suggested an association between high mRNA expression levels of *RAB31* and *SEC16B* and the resistance of various cancer cell lines to 62 and 17 of small molecule anti-cancer drugs, respectively. Furthermore, we link the expression levels of the hub genes and response to anti-cancer therapy using transcriptome-level data of the cancer patients. Interestingly, the high expression levels of *IRAK3*, *RAB31*, *LIN9*, and *OBSCN* predicted resistance of the cohort to chemotherapy. Moreover, these high expression levels of the hub genes also predicted worse patient outcomes to PD1, CTL4A, and PDL1 immunotherapies in the immune checkpoint blockage datasets.

Molecular docking is valuable in silico tool for modelling the possible interactions between a drug candidate and a target molecule [47], allowing for the depiction of the behavior of the drug candidate at the binding cavity of the receptor and elucidating the possible biological processes regulated by the drug candidate [48,49]. Interestingly, our molecular docking study suggested that the ovatodiolide docked well into the binding cavity of *RAB31, IRAK3, OBSCN, LIN9, TNPO2,* and *SEC16B* with estimated binding affinities ranging from 6.2–8.5 Kcal/mol. Non-covalent interactions such as hydrogen bonding, hydrophobic and ionic interactions, and Van der Waals forces play crucial roles in stabilizing the interaction between the drug candidate and protein targets [50]. Our docking analysis suggested that the interactions of ovatodiolide with the hub genes predominantly involved hydrogen bonds, Van der Waals forces, alkyl interaction, and various hydrophobic contacts (Figure 8, Table 3), having higher estimated binding affinities for the binding pockets of RAK3, SEC168, and *TNPO2* than it does for other genes.

The predicted Van der Waals forces around the ovatodiolide backbone with the respective amino acid of the receptors would likely create a strong cohesive environment, which could further stabilize the complexes [51]. Altogether, these molecular docking suggested that ovatodiolide has the molecular properties to interact with the binding site of *RAB31, IRAK3, OBSCN, LIN9, TNPO2,* and *SEC16B*. However, the higher numbers of interactions and binding affinities predicted between the ovatodiolide and RAK3, SEC168, and *TNPO2* suggested that the ovatodiolide would likely have higher druggable potentials against these genes than it does for *OBSCN*, *LIN9*, and *IRAK3*. Further in vitro and in vivo studies are ongoing in order to validate our in silico findings and to fully evaluate the therapeutic efficacy of this compound against the hub genes and cancers.

## 5. Conclusions

Collectively, our study suggested that the cancer hub genes, namely, *RAB31, IRAK3, OBSCN, LIN9, TNPO2,* and *SEC16B*, are crucial biomarkers of the immuno-oncology context of the tumor microenvironment, tumor staging, prognosis, and therapy response in liver, lung, stomach prostate, and colorectal cancer. Our results suggested that the cancer hub genes were associated with the suppressive nature of the TME and the poor prognosis of the patients. This cancer hub gene signature may serve as a valuable theranostic signature in the clinical setting upon further validation. On the translational forefront, our findings suggested that ovatodiolide could be an immunotherapeutic agent for the druggability of these hub genes.

## Figures and Tables

**Figure 1:**
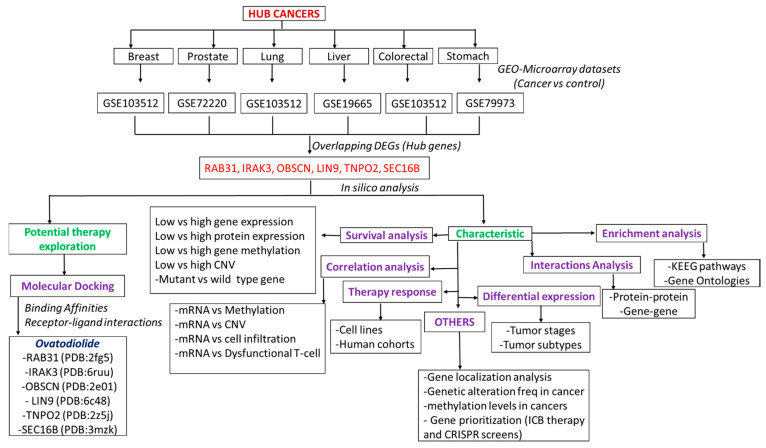
The flow chart of the study design for identifying the key DEGs in the pathology of the six cancers and predicting their potential druggability for ovatodiolide.

**Table 1:** Characteristics of datasets used for the identification of the differentially expressed genes (DEGs).

Cancers	Dataset		Platform	Tumor	Normal
Breast	GSE103512	GPL13158	[HT_HG-U133_Plus_PM] Affymetrix HT HG-U133+ PM Array Plate	65	10
Colorectal	GSE103512	GPL13158	[HT_HG-U133_Plus_PM] Affymetrix HT HG-U133+ PM Array Plate	57	12
Lung	GSE103512	GPL13158	[HT_HG-U133_Plus_PM] Affymetrix HT HG-U133+ PM Array Plate	60	9
Prostate	GSE72220	GPL5175	[HuEx-1_0-st] Affymetrix Human Exon 1.0 ST Array	57	90
Liver	GSE19665	GPL570	[HG-U133_Plus_2] Affymetrix Human Genome U133 Plus 2.0 Array	10	10
Stomach	GSE79973	GPL570	HG-U133_Plus_2] Affymetrix Human Genome U133 Plus 2.0 Array	10	10

**Table 2:** Distribution of data collection from the NCI Genomic Data Commons.

Cancers	mRNA Expression	Survival	Stage	CNV	SNV	Methylation
LUAD	578	481	473	517	567	528
BRCA	1220	1037	1034	1081	1026	816
STAD	452	373	341	442	439	416
PRAD	552	354	0	493	498	576
COAD	331	442	433	452	407	361
LIHC	426	197	176	371	365	453

CNV: copy number variation, SNV: single nucleotide variation.

**Table 3:** Docking profile of ovatodiolide with the cancer hub genes.

		Cancer Hub Genes				
Interactions	*IRAK3*	*LIN9*	*OBSCN*	*RAB31*	*SEC16B*	*TNPO2*
ΔG = (Kcal/mol)	−8.4	−6.5	−6.2	−6.5	−8.5	−8.1
Conventional H-bond	SER295 (2.92 Ӑ)	GLN401 (2.21 Ӑ)	GLN88 (2.50 Ӑ) ALA12 (3.77 Ӑ)	GLY15 (3.57 Ӑ)	ALA156 (2.33 Ӑ) GLN106 (3.34 Ӑ)	
π-alkyl	TYR340 ALA297	ILE396 ALA386 PHE400 LYS390 TYR404	LEU90	ILE34 PHE30	LEU264 PRO214 PRO155 PRO109	ALA380 ARG376 LEU419 LYS377
Van der waal forces	ASN298 ASP311 SER293 ILE294 LEU338 TRP339 HIS337 VAL373	ALA405 VAL408	ALA17 PRO16 GLN15 PHE11 VAL9 LYS13	LYS119 GLY17 HIS32 ASP31	TRP212 ALA108 TRP107 TRP153	ILE457 ARG495 TRP460 ARG464 ASP384 ALA423 ALA381
Hydrophobic Interaction (Ӑ)	ALA297 (3.68) HIS337 (3.71) TYR340 (3.80)	ILE396 (3.77) GLN401 (3.95) TYR404 (3.75)	LEU90A (3.70)	PHE30 (3.80)	PRO109 (3.70) PRO155 (3.60)	LEU419 (3.57) ALA423 (3.62) TRP460 (3.58)

## Data Availability

The datasets generated and analyzed in this study will be available upon reasonable request.

## Supplementary Materials

The following are available online at https://www.mdpi.com/article/10.3390/cancers13153847/s1. Table S1: Mutation frequency of the hub genes in the hub cancers, Table S2: Correlation of gene expression and tumor infiltration of the immune cells in hub cancer, Table S3: Correlation of gene expression and tumor infiltration of the immunosuppressive cells, Figure S1: Summary the associations between subtypes and gene expression, Figure S2: Kaplan–Meir plots survival differences between the mRNA expression levels of the hub genes in the combined cohorts of the hub cancer, Figure S3: Interaction map of the hub genes and pathway.
