# Supplementary material for: Identification of Cancer Hub Gene Signatures Associated with Immune-Suppressive Tumor Microenvironment and Ovatodiolide as a Potential Cancer Immunotherapeutic Agent
Source: Cancers (Basel). 2021 Jul 30;13(15):3847. doi: 10.3390/cancers13153847 (PMC8345223; doi:10.3390/cancers13153847)
Supplement: Supplementary file 1 [file cancers-13-03847-s001.zip › cancers-1274328-supplementary.pdf]

cancers

Supplementary Material: Identification of Cancer Hub Gene Signatures Associated with Immune-Suppressive Tumor Microenvironment and Ovatodiolide as a Potential Cancer Immunotherapeutic Agent

Jia-Hong Chen, Alexander T. H. Wu, Bashir Lawal, David T. W. Tzeng, Jih-Chin Lee, Ching-Liang Ho and Tsu-Yi Chao

Table S1. Mutation frequency of the hub genes in the hub cancers.

Cancer type	Symbol	EffectiveMut	NonEffectiveMut	Sample_Size	Percentage	Entrez
BRCA	*IRAK3*	5	3	1026	0.487329	11213
COAD	*IRAK3*	9	3	407	2.211302	11213
LIHC	*IRAK3*	4	1	365	1.09589	11213
LUAD	*IRAK3*	4	6	567	0.705467	11213
PRAD	*IRAK3*	5	1	498	1.004016	11213
STAD	*IRAK3*	8	5	439	1.822323	11213
BRCA	*LIN9*	7	4	1026	0.682261	286826
COAD	*LIN9*	5	3	407	1.228501	286826
LUAD	*LIN9*	7	0	567	1.234568	286826
PRAD	*LIN9*	1	0	498	0.200803	286826
STAD	*LIN9*	6	3	439	1.366743	286826
BRCA	*OBSCN*	47	30	1026	4.580897	84033
COAD	*OBSCN*	97	58	407	23.83292	84033
LIHC	*OBSCN*	38	19	365	10.41096	84033
LUAD	*OBSCN*	93	52	567	16.40212	84033
PRAD	*OBSCN*	16	11	498	3.212851	84033
STAD	*OBSCN*	83	60	439	18.90661	84033
BRCA	*RAB31*	2	1	1026	0.194932	11031
COAD	*RAB31*	3	0	407	0.737101	11031
LIHC	*RAB31*	2	0	365	0.547945	11031
LUAD	*RAB31*	3	1	567	0.529101	11031
BRCA	*SEC16B*	15	7	1026	1.461988	89866
COAD	*SEC16B*	11	9	407	2.702703	89866
LIHC	*SEC16B*	2	1	365	0.547945	89866
LUAD	*SEC16B*	14	3	567	2.469136	89866
PRAD	*SEC16B*	3	1	498	0.60241	89866
STAD	*SEC16B*	5	1	439	1.138952	89866
BRCA	*TNPO2*	5	4	1026	0.487329	30000
COAD	*TNPO2*	10	6	407	2.457002	30000
LIHC	*TNPO2*	2	1	365	0.547945	30000
LUAD	*TNPO2*	5	2	567	0.881834	30000
PRAD	*TNPO2*	1	0	498	0.200803	30000
STAD	*TNPO2*	12	4	439	2.733485	30000

Table S2. Correlation of gene expression and tumor infiltration of the immune cells in hub cancer.

Cancer	Variable	*RAB31*	*IRAK3*	*OBSCN*	*LIN9*	*TNPO2*	*SEC16B*
BRCA	Purity	-0.07114	-0.39882	-0.0793	0.138495	0.11875	-0.22121
BRCA	B Cell	0.028272	0.153182	-0.08747	0.17233		
BRCA	CD8+ T Cell	0.206092	0.430456	-0.16596	0.198325	0.112571	0.069031
BRCA	CD4+ T Cell	0.128787	0.366357	0.198201	0.136632	0.20206	0.270966
BRCA	Macrophage	0.424807	0.376069	-0.10949			0.177569
BRCA	Neutrophil	0.222228	0.397411		0.209792	0.172728	0.132569
BRCA	Dendritic Cell	0.197187	0.363863		0.151738	0.09814	0.089549
COAD	Purity	-0.40478	-0.32391		0.098922		0.10333
COAD	B Cell	0.173817	0.256238	-0.1289	0.237989		0.120667
COAD	CD8+ T Cell	0.381718	0.389297		0.302618	-0.12165	

COAD	CD4+ T Cell	0.435098	0.422662	0.110091		0.434489	0.278879
COAD	Macrophage	0.70418	0.535847	0.176062	0.185667	0.111776	
COAD	Neutrophil	0.651601	0.56247		0.264135		
COAD	Dendritic Cell	0.653389	0.548536		0.178367	0.112063	
LIHC	Purity	-0.49643	-0.45706	-0.21289	0.216315		0.11
LIHC	B Cell	0.421792	0.250275	0.278656	0.387734	0.237761	
LIHC	CD8+ T Cell	0.484147	0.338181	0.208561	0.248836	0.16781	-0.17703
LIHC	CD4+ T Cell	0.5219	0.431311	0.474799	0.460894	0.501213	
LIHC	Macrophage	0.681213	0.478544	0.474776	0.44294	0.417728	-0.10642
LIHC	Neutrophil	0.584799	0.565076	0.299182	0.408033	0.382461	
LIHC	Dendritic Cell	0.662407	0.450874	0.283084	0.397766	0.334517	
LUAD	Purity	-0.26168	-0.27775		0.133162		-0.0932
LUAD	B Cell	-0.02583	0.307063		-0.18291		
LUAD	CD8+ T Cell	0.266995	0.201114	-0.18584	0.145957		-0.09153
LUAD	CD4+ T Cell	0.102133	0.299907	0.232341	-0.1754	0.306938	0.269201
LUAD	Macrophage	0.350957	0.312079				
LUAD	Neutrophil	0.452357	0.449962		0.109917	0.141522	
LUAD	Dendritic Cell	0.390715	0.401976				
PRAD	Purity	-0.45332	-0.4882	-0.28497			
PRAD	B Cell	0.596777	0.494273	0.155744	0.450827	0.387525	0.291824
PRAD	CD8+ T Cell	0.484187	0.635431		0.412507	0.441604	0.318523
PRAD	CD4+ T Cell	0.520793	0.371931	0.358228	0.184186	0.133199	0.179781
PRAD	Macrophage	0.651215	0.612966	0.223254	0.326646	0.384702	0.267597
PRAD	Neutrophil	0.618639	0.571184	0.3201	0.402695	0.373526	0.341577
PRAD	Dendritic Cell	0.729857	0.611958	0.141348	0.352691	0.329695	0.283255
STAD	Purity	-0.21219	-0.14336		0.188238	0.112129	
STAD	B Cell	-0.1376	0.06274	0.180881			0.131509
STAD	CD8+ T Cell	0.266926	0.305153	-0.11899	-0.1689		
STAD	CD4+ T Cell	0.356594	0.205684	0.261254	-0.11785	0.231008	0.146137
STAD	Macrophage	0.704372	0.318358		-0.2204		
STAD	Neutrophil	0.415955	0.342417	-0.13586	-0.11733		
STAD	Dendritic Cell	0.550791	0.345729		-0.19096		

Table S3. Correlation of gene expression and tumor infiltration of the immunosuppressive cells.

**CAF**	***RAB31***	***IRAK3***	***0BSCN***	***LIN9***	***TNPO2***	***SEC16B***
BRCA	0.555	0.321	0.14	-0.219	-0.128	0.254
COAD	0.945	0.5114	0.145	-0.139	0.24	-0.138
LIHC	0.701	0.523	0.266	0.184	0.335	
LUAD	0.541	0.132		-0.291	0.283	
PRAD	0.78	0.694	0.126	0.225	0.22	0.137
STAD	0.863	0.166	0.162	-0.343	0.152	-0.218

MDSC	RAB31	IRAK3	0BSCN	LIN9	TNPO2	SEC16B
BRCA	-0.186	-0.305		0.424	0.346	-0.232
COAD	-0.283	-0.349		0.23	0.13	-0.158
LIHC	0.204		0.352	0.58	0.398	-0.271
LUAD				0.44	0.273	-0.121
PRAD	-0.233	-0.358	-0.147	0.174	0.127	-0.101
STAD	-0.305	-0.351	0.123	0.353	0.059	-0.16

TAM	RAB31	IRAK3	0BSCN	LIN9	TNPO2	SEC16B
BRCA	-5.993	-3.2		-0.27		-0.01
COAD	-5.24	-3.65				-0.01
LIHC	-4.95	-2.90			-0.14	-0.04
LUAD	-5.90	-3.02		-0.136	-0.18	-0.01
PRAD	-6.04	-2.93	-2.54	-0.199	-0.23	-0.146
STAD	-5.01	-3.34	0.15	-0.213	-0.19	-0.179

TREG	RAB31	IRAK3	OBSCN	LIN9	TNPO2	SEC16B
BRCA	0.238	0.282	0.245	0.166	0.186	0.152
COAD	0.487	0.415		0.123	0.287	0.19
LIHC	0.453	0.405	0.361	0.416	0.369	0.236
LUAD	0.387	0.525	0.166		0.257	0.123

PRAD	0.504	0.473	0.209	0.359	0.317	0.224
STAD	0.365	0.364	0.247	0.104	0.309	0.217

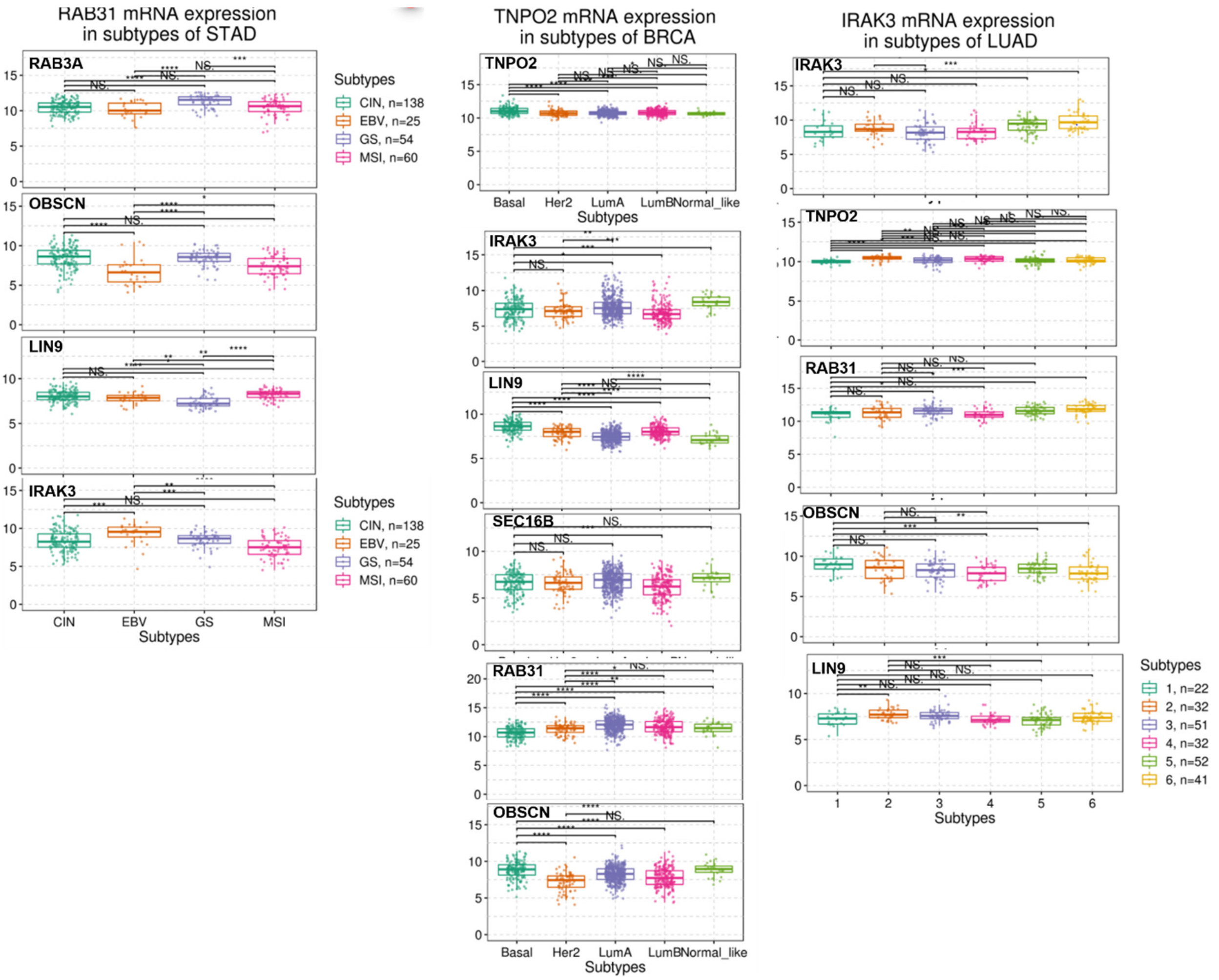

Figure S1. Summary the associations between subtypes and gene expression.

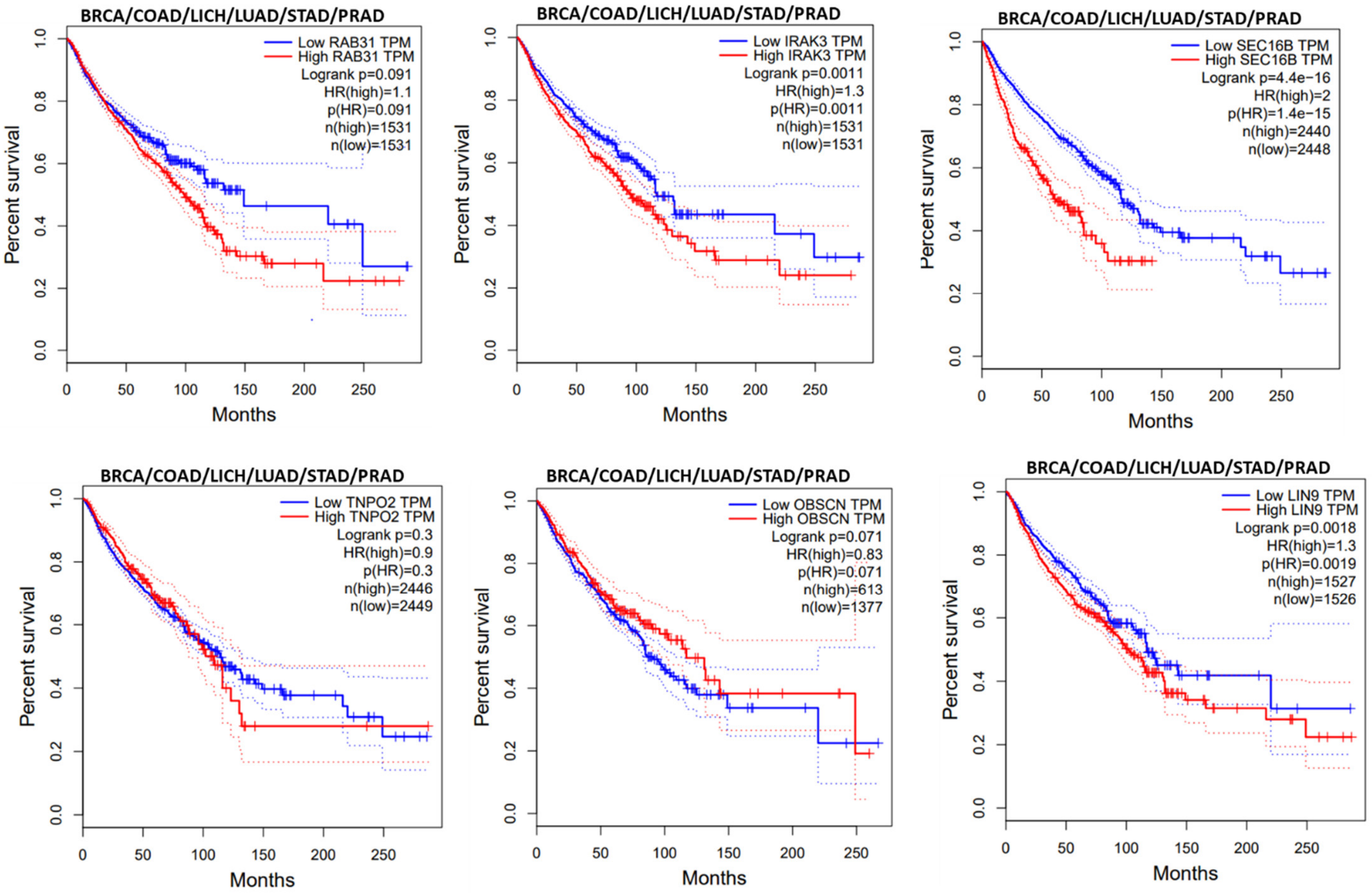

Figure S2: Kaplan–Meir plots survival differences between the mRNA expression levels of the hub genes in the combined cohorts of the hub cancer.

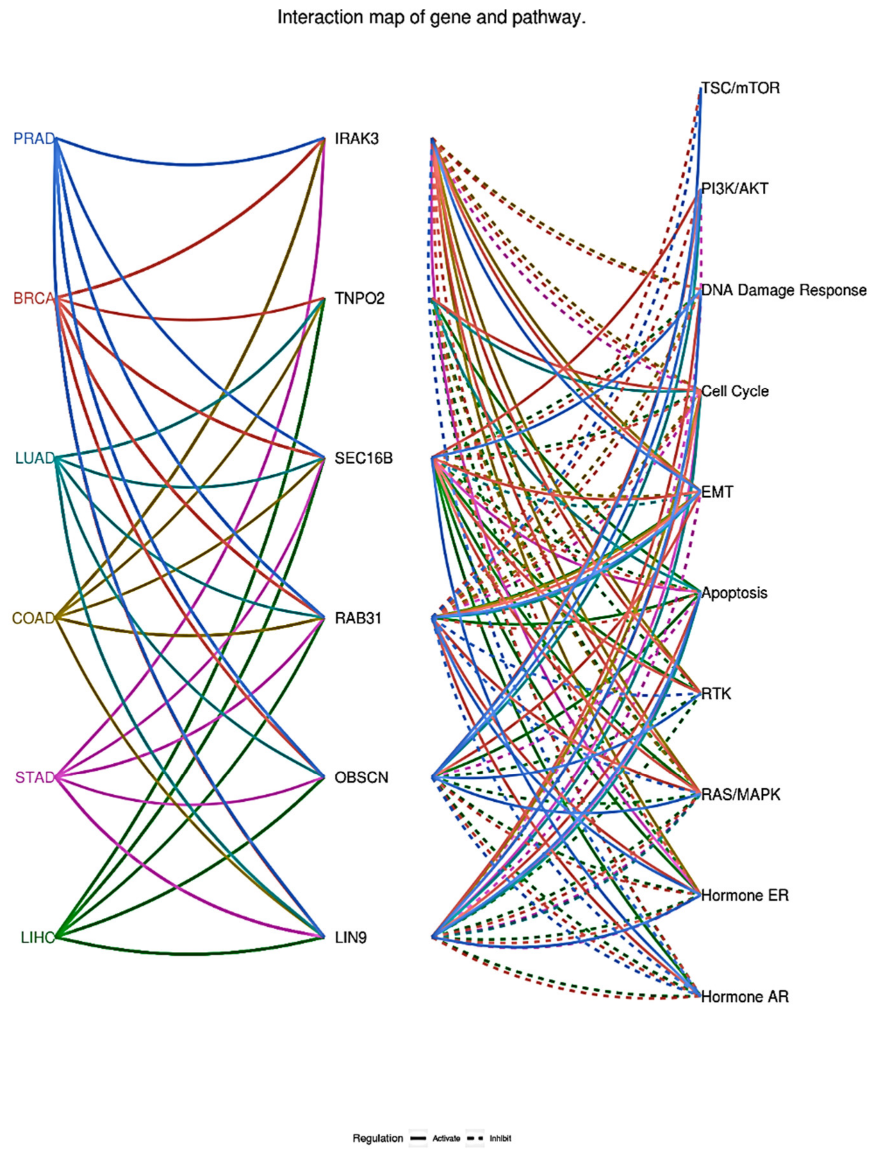

Figure S3. Interaction map of the hub genes and pathway.
